# The Mechanism of Deformation Compatibility of TA2/Q345 Laminated Metal in Dynamic Testing with Split-Hopkinson Pressure Bar

**DOI:** 10.3390/ma16247659

**Published:** 2023-12-15

**Authors:** Yanshu Fu, Shoubo Chen, Penglong Zhao, Xiaojun Ye

**Affiliations:** 1 School of Advanced Manufacturing, Nanchang University, Nanchang 330031, China; yshfu@ncu.edu.cn (Y.F.); 13767030491@163.com (S.C.); yexiaojun@ncu.edu.cn (X.Y.); 2 School of Information and Artificial Intelligence, Nanchang Institute of Science & Technology, Nanchang 330108, China

**Keywords:** deformation compatibility, stress-state equilibrium, laminated metal

## Abstract

The laminated metal materials are widely used in military, automobile and aerospace industries, but their dynamic response mechanical behavior needs to be further clarified, especially for materials with joint interface paralleling to the loading direction. The mechanical properties of TA2/Q345 (Titanium/Steel) laminated metal of this structure were studied by using the split Hopkinson pressure bar (SHPB). To shed light on the stress-state of a laminated metal with parallel structure, the relative non-uniformity of internal stress *R*(*t*) was analyzed. The mechanism of deformation compatibility of welding interface was discussed in detail. The current experiments demonstrate that in the strain rate range of 931–2250 s^−1^, the discrepancies of the internal stress in specimens are less than 5%, so the stress-state equilibrium hypothesis is satisfied during the effective loading time. Therefore, it is reasonable to believe that all stress–strain responses of the material are valid and reliable. Furthermore, the four deformation stages, i.e., the elastic stage, the plastic modulus compatible deformation stage, uniform plastic deformation stage and non-uniform plastic deformation stage, of the laminated metal with parallel structure were firstly proposed under the modulating action of the welding interface. The deformation stages are helpful for better utilization of laminated materials.

## 1. Introduction

Laminated metal materials are widely used in many applications such as military, automobile and aerospace industries due to a number of unique combinations of high physical, mechanical and operational properties [1,2,3,4]. The laminated materials can consist of different kinds of layers such as ceramic/metal, metal/metal, and metal/composite and so on. Studies have shown that the multilayered composite structure is lighter in weight, has higher impact resistance, and more designable than homogeneous materials [5,6]. Fernando et al. [7] presented a comprehensive analysis of the blast response of functionally graded composite metallic plates and observed that the impedance graded composite plates, which were lighter in density than the monolithic plates, resisted the highly intensive blast loads through their enhanced ductility. Gladkovsky et al. [8] researched the microstructure and mechanical properties of sandwich copper/steel composites materials. They pointed out that the composites have higher strength properties than initial cooper by approximately 1.8–3.5 times. Besides the quasi-static compression tests, the dynamic responses are also different between them [9]. As for composites or laminated materials, it is essential to understand their mechanical behavior and constitutive model under wide strain rate loading [10,11]. Zhang et al. [12] employed bumpers constructed from Impedance-graded materials (IGM) to improve meteoroid/debris shielding structures for spacecrafts. In their research works, the wave propagation and thermodynamic states in the bumper materials were discussed, and it was shown that the impedance-graded bumper changes the wave propagation path and duration time, which lead to enough time to make materials very hot and break up, and the solid projectile fragments are expanded over a greater area. 

The split Hopkinson pressure bar (SHPB) technique is the most classical method for obtaining response properties of materials at dynamic compressions [13,14,15,16,17]. It was developed for detecting explosive waves by Hopkinson and developed revolutionarily by Kolsky in 1949 for obtaining properties of materials at high strain rates [18,19]. It is worth to note that the dissimilar materials with differing moduli and impedances will cause complex wave reflection and transmission phenomena at each encountered interface [20]. The impedance represents the resistance a material presents to the transmission of stress waves, and it is a product of the material’s density and wave propagation speed. The stress wave impedance mismatch refers to the mismatch in the acoustic or mechanical impedance between two materials when a stress wave propagates from one material to another. When a vertical or oblique, but not parallel, stress wave is incident and encounters an interface between two materials with different impedance values, some of the wave’s energy can be reflected back and some can be transmitted to the other material. The extent of reflection and transmission depends on the difference in impedance between the materials. If the impedance mismatch is significant, a large portion of the wave’s energy can be reflected, leading to poor transmission of stress and potentially causing issues such as energy loss, reduced signal quality, or even structural damage. Hui et al. [1] pointed out that the wave propagation of shock wave’s reflection behavior in an IGM bumper has led to better attenuation of shock wave energy by multiple interface reflections and transmissions, which should play an important role in the heating and fracture effect of bumpers and projectiles.

As for laminated materials, the mechanism of deformation compatibility under dynamic impact loading is very important. However, the research works mentioned above only paid attention to the behavior of laminated materials when the impact loaded vertically to the welding interface. They lacked a study of the effect of the impact loading component, which is parallel to the welding interface. Actually, when impact loads are applied to laminated materials, the vertical and parallel components almost always accompany each other, so it should be necessary to detect the behavior of laminated materials responding to the parallel structure of the multi-layer interfaces. There are, as of yet, no experimental, numerical or analytical studies available in the published literature. This present work was initially motivated by a need to address the research gap of the lack of comprehensive studies for multi-layered metal plates responding to the impact loading parallel component of the multi-layer interfaces. As such, experimental and analytical studies were conducted for an impedance mismatch composite metal plate using the SHPB test system and mathematical model. 

The remainder of this paper is organized as follows. In Section 2, we describe the configurations of the experimental setup and the specimen preparations in detail. Then, we present a series of analyses concerning the design of the experiments, the mode-mix analysis, and the post-processing of the raw data for extraction of the mixed-mode properties as well as a complete set of results in Section 3, where the effects of the separation rate and mode-mix are discussed at length. Conclusions are provided in Section 4.

## 2. Material and Methods

### 2.1. Experimental Setup

**Figure 1:**
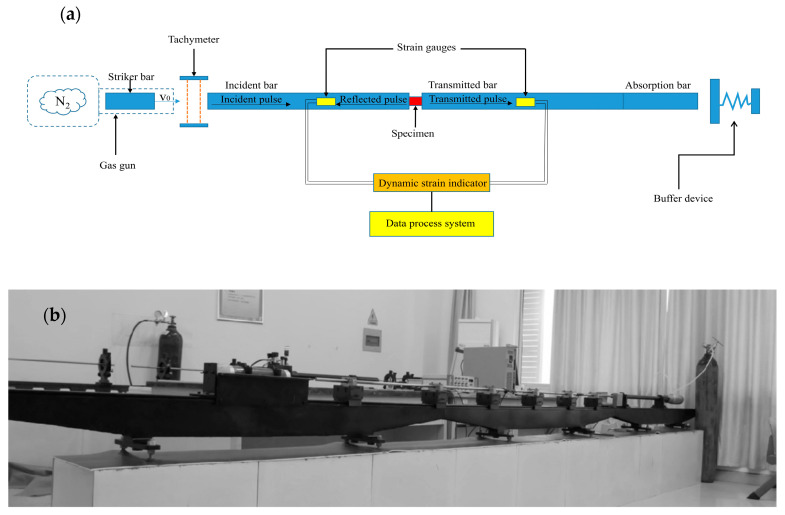
Schematic of SHPB (**a**) schematic diagram, and (**b**) experimental apparatus.

A conventional SHPB apparatus is shown in Figure 1, consisting of a gas gun, a projectile or striker bar (φ14.5 × 200 mm), an incident bar (φ14.5 × 1000 mm), a transmitted bar (φ14.5 × 1000 mm), an energy absorber bar (φ14.5 × 600 mm) and a high-frequency data processing system. The specimen was placed between the incident and transmitted bars. As the striker bar hits the incident bar at a constant speed *v*_0_, an incident wave is generated and transmitted throughout the bar. When it propagates to the interface between the incident bar and specimen, the incident wave will reflect and transmit due to the different acoustic impedances of the contacted materials which is called acoustic impedance mismatch hereinafter. Part of the incident wave becomes a reflected wave and transfers towards the incident bar with velocity in the opposite direction, while the other part wave becomes a transmission wave and propagates through the specimen. The amounts of reflected and transmitted waves at the interfaces depend on the mechanical impedance ratio of the bars and the specimen. Soon, at the specimen/transmitted bar interfaces, the reflection and transmission phenomenon happened again. Eventually, the energy of the transmitted wave will be dissipated in the absorber bar.

It is necessary to ensure the bars in the apparatus are always keeping linear elasticity during the experiments, and the length of the bar must be far larger than the diameter to ignore the transverse inertial effect, so that the data can be processed based on the one-dimensional stress wave theory. The strain rate $\dot{\varepsilon }$, nominal strain *ε*, and the nominal stress σ are given as follows:

(1) $$\dot{\varepsilon }\left(t\right)=\frac{C_{0}}{L_{s}}\left(\varepsilon_{i}-\varepsilon_{r}-\varepsilon_{t}\right)$$

(2) $$\varepsilon (t)=\frac{C_{0}}{L_{s}}\int_{0}^{t}\left(\varepsilon_{\;i}-\varepsilon_{r}-\varepsilon_{\;t}\right)dt$$

(3) $$\sigma (t)=\frac{A}{2A_{s}}E(\varepsilon_{\;i}+\varepsilon_{r}+\varepsilon_{\;t})$$

where *C*_0_, *A* and *E* are the wave speed, cross-section area and the elastic modulus of the bar, respectively. *A_s_* and *L_s_* are the initial cross-section area and length of the specimen. $\varepsilon_{i}$, $\varepsilon_{r}$ and $\varepsilon_{t}$ are the incident strain wave, the reflected strain wave and the transmitted strain wave, respectively.

Assuming that the specimen deforms uniformly in longitudinal direction, then

(4) $$\varepsilon_{i}+\varepsilon_{r}=\varepsilon_{t}$$

Substituting Equation (4) into Equations (1)–(3), we obtain:

(5) $$\dot{\varepsilon }(t)=-\frac{2C_{0}}{L_{s}}(\varepsilon_{i}-\varepsilon_{t})=-\frac{2C_{0}}{L_{s}}\varepsilon_{r}$$

(6) $$\varepsilon (t)=-\frac{2C_{0}}{L_{s}}\int_{0}^{t}\left(\varepsilon_{i}-\varepsilon_{t}\right)dt=-\frac{2C_{0}}{L_{s}}\int_{0}^{t}\left(\varepsilon_{r}\right)dt$$

(7) $$\sigma (t)=\frac{A}{A_{s}}E(\varepsilon_{\;i}+\varepsilon_{\;r})=\frac{AE}{A_{s}}\varepsilon_{\;t}$$

By using Equations (5)–(7), the stress–strain curves corresponding to series of strain rates can be deduced.

In this study, the stress signals were measured by the strain gauges that attached on the incident and reflected bars. The sensitivity of the gauge is 1 V/1000 μ*ε*, and the range is 0~±3 × 10^4^ μ*ε*. The movement of the striker is controlled by high-pressure Nitrogen, with an adjustment accuracy of 0.01 MPa. The striker bar velocities are 6.31 m/s, 13.63 m/s, 16.73 m/s, 19.24 m/s, and 22.03 m/s, denoted as cases 1–5 hereafter, as shown in Table 1.

### 2.2. Specimen Preparations

The laminated metal is fabricated using explosive welding of 30 mm-thick base steel material Q345 and 8 mm thick composite titanium material TA2. Explosive welding, belonging to solid-state welding, has been used to connect a wide range of dissimilar impedance materials and obtain firm bonds through high pressure and heat without causing significant crystallization or phase transition [21]. The metal performs act as an elastic-viscous plastic fluid under the detonation wave. The type of explosive is rock ammonium nitrate, with a charge density of 0.8 g/cm^3^. The explosive detonates at a speed of 2800 m/s, and the gap between the basic plate (Q345) and the fly plate (TA2) is about 6 mm. Under the proper welding parameters, the repeatability of the laminated materials could be ensured.

**Figure 2:**
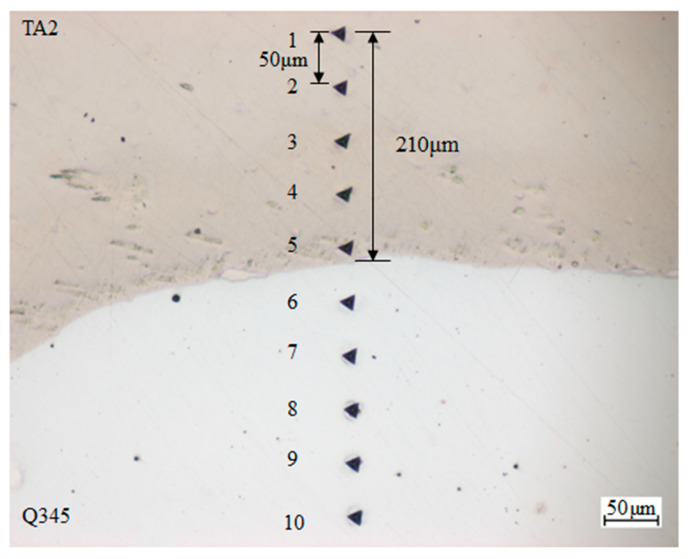
Nano-indentation test of TA2/Q345 laminated metal (The numbers 1 to 10 correspond to ten test points).

**Figure 3:**
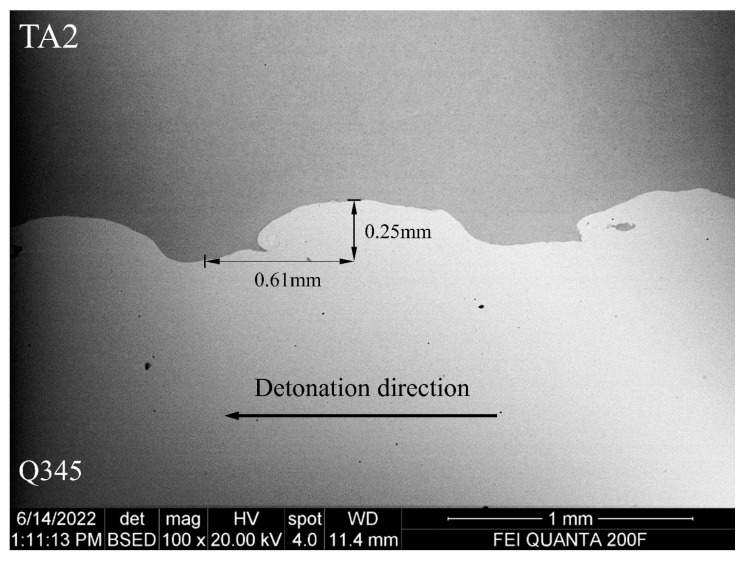
Microstructure of TA2 and Q345 welding interface.

Different from the pure Q345 and TA2, the properties of these two metals are changed after explosive welding. The elasticity modulus of Q345 and TA2 in laminated materials is tested with nano-indentation of iMicor (shown in Figure 2). The tests points were arranged from TA2 to Q345 at 210 μm from the welding interface with a spacing of 50 μm. The elasticity modulus is averaged with 5 test points, respectively. The properties of Q345 and TA2 as well as the SHPB bars are listed in Table 2. The microstructure of TA2/Q345 welding interface is shown in Figure 3. It can be seen that there exists a wavy interface at the steel and titanium boundary with no obvious defects. The wavy interfaces ensure that the laminated metal has favorable mechanical properties and a strong bond area. It is clear from Figure 3 that twice the amplitude and half the wavelength are 0.25 mm and 0.61 mm, respectively. Since the wavelength is almost ten times the amplitude, the welding interface can be regarded as a plane macroscopically for the sake of simplicity.

**Figure 4:**
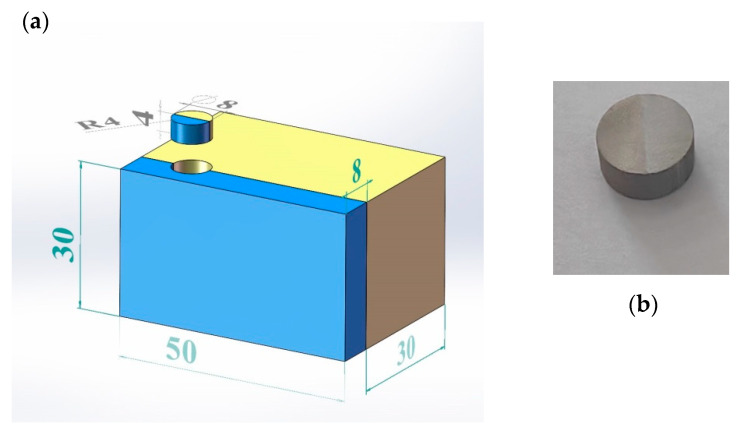
Geometrical parameter of the TA2/Q345 and specimen (**a**) and specimen diagram (**b**).

As previously mentioned, the initial assumption in the wave analysis of the SHPB is the stress-state equilibrium in the specimen, meaning that the force on the incident bar side of the specimen is equal to that on the transmitted bar side of the specimen [22,23,24,25]. In order to achieve the required stress-state equilibrium and stress level in the specimen, the length of the specimen should be greater than the transit time for the stress pulse in the specimen, and the cross-section area of the specimen should be kept smaller than the bar. For trial and error, the specimen is designed a cylinder with a diameter of 8 mm and a length of 4 mm. The geometrical parameter of the specimen and the sampling method are shown in Figure 4a. The original welding TA2/Q345 laminated metal is about 400 mm × 300 m × 38 mm. In order to process the specimen, a cube of 30 mm × 50 mm × 38 mm material is cut off first. Then, a ϕ8 × 4 mm cylinder was made through wire electrical discharge technology (Figure 4b). The two ends of the specimen are polished with sanding to ensure their parallelism is within the tolerance range of 0.01 mm. The weight of the specimen is 1.24 g, and it was placed between the incident and transmitted bars with Vaseline. Vaseline was used to reduce the friction on both end faces of the specimen and to help keep the specimen contacting with the bars at the center line of the bars. As can be seen, the welding interface is located in the middle, and the volume fraction of Q345 and TA2 accounts for 50% each. In this research, we define samples as parallel samples, meaning that the interface parallels the loading direction, so the stress wave propagates along the interface. Because of the physical and mechanical properties differences between Q345 steel and Ti, the wave impedance is different from each other, but they were combined together by explosive welded technology, so samples in this work are made from impedance-mismatched multilayered materials. (All devices, instruments, and materials are used or made in Nanchang City, Jiangxi Province, China).

## 3. Results and Discussion

### 3.1. Typical Waveform of SHPB Tests of TA2/Q345

**Figure 5:**
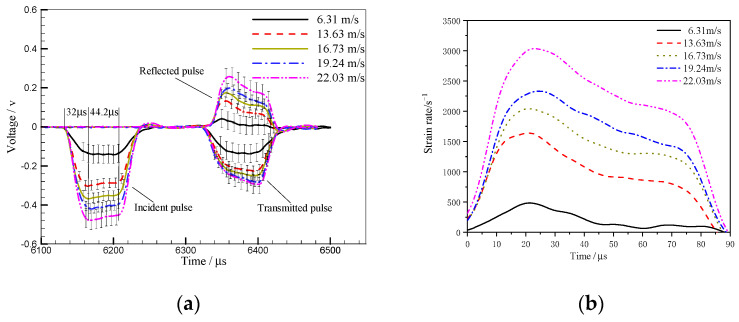
The stress-time curves (**a**) and stress rate–time curves (**b**) of cases 1–5.

In the experiments, the stress wave propagation characteristics of the parallel specimen were obtained at different impact loads controlled by the velocity of the striker bar. Each case was repeated three times, and the stress-time as well as the strain rate–time curves of the specimens are shown in Figure 5. The averaged values of the repeated experiments corresponding to the same case were presented with an error bar to show the statistical analyses of the achieved results.

As shown in Figure 5a, the rise time of an incident wave is ~32 μs and the corresponding platform segment after reaching the maximum value lasts about 44.2 μs. As for the reflected wave, it declines gradually over time after rising to the peak, indicating the high-work-hardening rate of TA2/Q345 laminated metal materials [26]. Since the shape and amplitude of the transmitted wave are determined by the specimen’s stress–strain behavior, the gradual increase of the transmitted wave’s amplitude in Figure 5a indicates the strain-hardening response of TA2/Q345.

The strain rate can be calculated from the reflected pulse according to Equation (5) and the strain rate–time curves of cases 1–5 are plotted in Figure 5b. The strain-rate is not constant during the loading process as the reflected wave does not display constant amplitude in Figure 5a. This phenomenon is closely related to the material characteristics of TA2/Q345. The effective loading duration of incident wave is 32–76.2 μs. Therefore, the average strain rate of the stage is regarded as the mean strain rate in the whole loading process. In this study, the strain rates corresponding to cases 1–5 are 146 s^−1^, 931 s^−1^, 1384 s^−1^, 1686 s^−1^ and 2250 s^−1^.

### 3.2. Verification of No-Slip Condition at Welding Interface

During the experiment, the specimen was deformed under the action of dynamic compression load. In the deformation process, Q345 and TA2 have the same strain, i.e., no slip at the interface during the deformation process. No slip at the interface means that within a certain strain rate range, the welding interface will not be damaged; Q345 and TA2 are bound tightly, and Q345 and TA2 are co-deformed under the action of the welding interface. In order to verify whether the condition of no slip at the interface during the impact is established, the specimen is cut axially after impact, and the state of the welding interface is observed under Axioscope optical microscope after grinding and polishing to investigate whether slip damage occurs.

**Figure 6:**
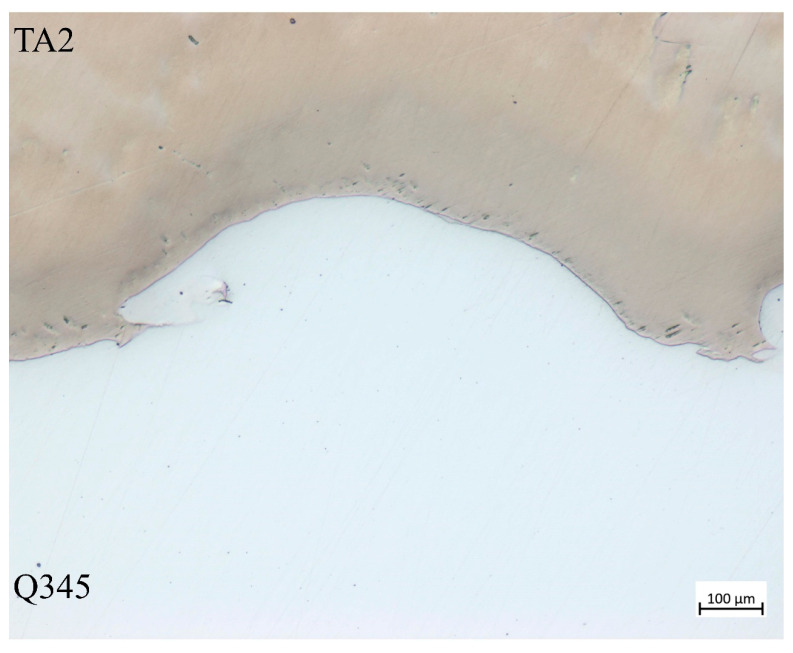
The microstructure of the welding interface of case 4.

**Figure 9:**
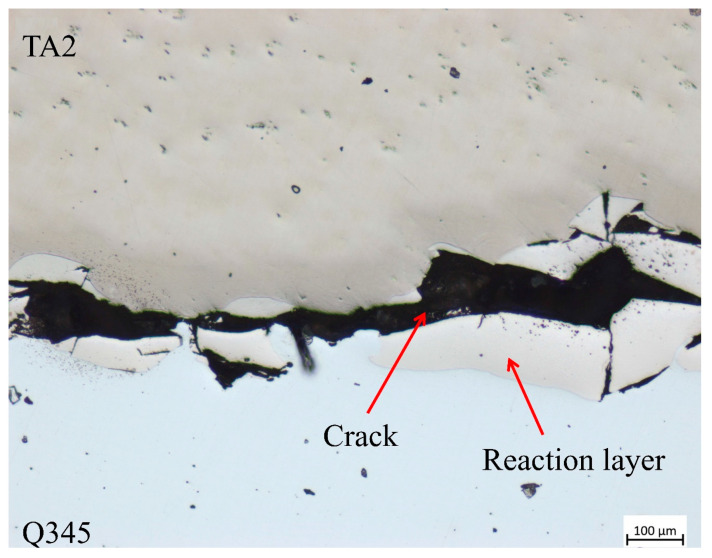
The microstructure of the welding interface of case 5.

Figure 6 shows the microstructure of the welded interface of case 4. It can be seen from Figure 6 that the bonding quality of the welded interface is still good at this strain rate. The phenomenon observed under dynamic loading conditions with strain rate lower than 1686 s^−1^ is consistent with Figure 6. Therefore, it can be inferred that the condition of no slip at the interface is valid in the range of strain rate from 0 to 1686 s^−1^. The damaged interface of case 5 is shown in Figure 9, which will be discussed in detail later.

In addition, the axial plastic deformation compression of the specimen is 0.49 mm of case 4. This indicates that the welding interface failure must occur after the specimen has undergone plastic deformation, and it can be inferred that the bonding strength of the welding interface is greater than the yield strength of Q345 and TA2, respectively.

### 3.3. Verification of the Specimen Stress Uniformity

According to the data processing principle of SHPB, it can be seen that the stress balance at both ends of the specimen in the experiment is sufficient and necessary to ensure the real and reliable experimental results. Therefore, it is very important to verify whether the specimen achieves stress uniformity quickly during loading. At present, it is generally accepted that when the stress wave is reflected back and forth in the specimen for two or three times, if the difference between the stress at both ends of the specimen and the ratio of its average value (in this case, the average value of the stress at both ends of the specimen is used as the average stress of the specimen) is less than 5%, the uniformity hypothesis can be considered satisfied [27]. However, because the material used in this paper is a TA2/Q345 laminate material, there is a large difference in material properties between the substrate (Q345) and the composite (TA2). Therefore, it is necessary to examine the stress state at both ends of the specimen during the experiment and evaluate the degree of stress uniformity inside the specimen to judge whether the experiment meets the uniformity hypothesis. According to the one-dimensional stress wave theory:

(8) $$\sigma_{t}^{L}=\frac{EA}{A_{s}}\left(\varepsilon_{i}+\varepsilon_{r}\right)$$

(9) $$\sigma_{t}^{R}=\frac{EA}{A_{s}}\varepsilon_{t}$$

where $\sigma_{t}^{L}$ is the stress at the left end of the specimen at time *t* (the end of the specimen in contact with the incident bar is the left end) and $\sigma_{t}^{R}$ is the stress at the right end of the specimen at the same time (the end of the specimen in contact with the transmission bar is the right end). Therefore, the stress difference between the two ends of the specimen $\Delta \sigma_{t}$ and the average stress of the specimen $\bar{\sigma_{t}}$ at time *t* are:

(10) $$\Delta \sigma_{t}=\sigma_{t}^{L}-\sigma_{t}^{R}$$

(11) $$\bar{\sigma_{t}}=\frac{\sigma_{t}^{L}+\sigma_{t}^{R}}{2}$$

The relative non-uniformity of the internal stress of the specimen at time *t* is defined as *R*(*t*) [28]:

(12) $$R(t)=\left|\frac{\Delta \sigma_{t}}{\bar{\sigma_{t}}}\right|$$

By substituting Equations (8)–(11) into Equation (12), we get:

(13) $$R(t)=\left|\frac{2\left(\varepsilon_{I}+\varepsilon_{R}-\varepsilon_{T}\right)}{\varepsilon_{I}+\varepsilon_{R}+\varepsilon_{T}}\right|$$

According to the existing evaluation criteria, if *R*(*t*) < 5% during the effective loading time of incident wave (incident wave platform segment), the experiment can be considered to meet the uniformity hypothesis and the experimental results are valid.

**Figure 7:**
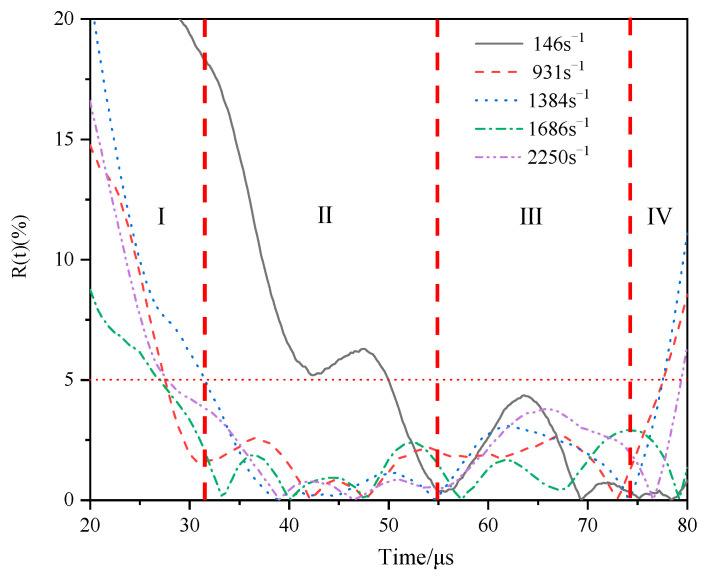
The variation of *R*(*t*) with time of cases 1–5.

In this paper, the variation of *R*(*t*) with time under different strain rate loading conditions is shown in Figure 7. As can be seen from Figure 7, for cases 2–5, *R*(*t*) of the effective loading time of the incident wave (as can be seen from Figure 5a that the effective loading time of the incident wave ranges from 32 μs to 76.2 μs) is less than 5%. Therefore, the experimental results are consistent with the assumption of uniformity, the experimental scheme is reasonable, and the experimental results are valid. However, as for case 1, *R*(*t*) is less than 5% when it is around 50 μs, and the uniformity hypothesis is satisfied only in the latter half of the effective loading time of the incident wave. Therefore, the stress–strain relationship derived from the reflected and transmitted waves measured for case 1 is not accurate. The material properties of TA2/Q345 laminates at this strain rate cannot be reflected correctly.

As shown in Figure 7, during the experiment, the changes of *R*(*t*) corresponding to cases 2–5 showed four different characteristics with time in turn. The *R*(*t*) − *t* curve can therefore be divided into four regions. They are I zone, II Zone, III zone and IV zone. In zone I, *R*(*t*) is always larger than 5%, which indicates that the stress state in the specimen during this process is not uniform. In zone II, *R*(*t*) is always less than 5% (except for *R*(*t*) in case 1), and remains around 2% after 40 μs. It indicates that the internal stress uniformity of the specimen remains at a high level during this period of time.

After entering the III zone, although *R*(*t*) is still less than 5%, significant fluctuation occurs, and the stress uniformity becomes worse. It is shown that the compatibility ability of the welding interface becomes worse under continuous stress wave loading. The fluctuation phenomenon appeared in cases 2–5, but only the initial time of the fluctuation was slightly different. There may be two reasons for the fluctuation: one is that the welding interface is damaged at this stage, resulting in the non-slip condition of the interface no longer being satisfied; the second reason is that the transverse deformation of the specimen accumulates to a certain extent at this stage, which intensifies the transverse inertia effect. According to Section 3.2, under the loading condition of strain rate from 0 to 1686 s^−1^, the welding interface is still valid, indicating that the fluctuation is mainly caused by the transverse inertia effect. In region III, *R*(*t*) is always less than 5%, indicating that the transverse inertia effect is not obvious. It also shows that the specimen satisfies the one-dimensional stress hypothesis during loading. The starting time of the data fluctuation in *R*(*t*) in region IV is around 75 μs. Combined with Figure 5a, it can be seen that the incident wave begins to unload at 76.2 μs. Therefore, the reason for the fluctuation of data in region IV is the unloading of the incident wave.

### 3.4. Four Deformation Stages during the Dynamic Response Process

According to the analysis of various regions of the *R*(*t*) − *t* curve in Figure 7 in the previous section, combined with the one-dimensional stress wave theory, it can be inferred that TA2/Q345 laminated specimens will enter the four deformation stages successively under the action of dynamic compression load under the mechanism of coordinated deformation participation of the welding interface. These are: the respectively elastic deformation stage, the plastic modulus compatibility deformation stage, the uniform plastic deformation stage and the non-uniform plastic deformation stage. 

#### 3.4.1. Elastic Deformation Stage

**Figure 8:**
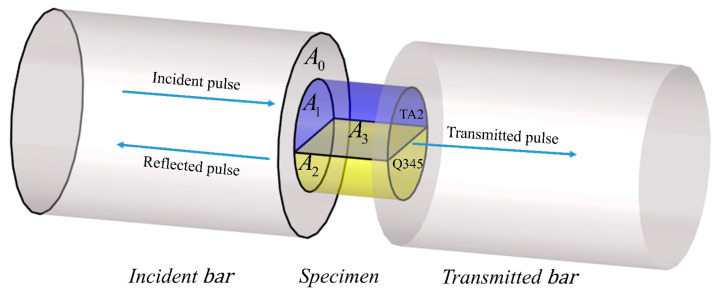
Loading diagram of TA2/Q345parallel specimen.

As shown in Figure 8, *A*_0_, *A*_1_, and *A*_2_ are the cross-sectional areas of the incident bar, TA2 and Q345, respectively. *A*_3_ is the area of the welding interface. According to Newton’s third law, the initial stress state of the contact surface between the incident bar and the specimen is:

(14) $$\sigma_{b}A_{0}=\rho_{1}c_{1}v_{1}A_{1}+\rho_{2}c_{2}v_{2}A_{2}$$

(15) $$\sigma_{b}=E\varepsilon_{i}$$

where, $\rho_{1}c_{1}$ and $\rho_{2}c_{2}$ are the wave impedance of TA2 and Q345, $\rho_{1}$ is the density of TA2, $\rho_{2}$ is the density of Q345, *c*_1_ is the elastic wave velocity of TA2, *c*_2_ is the elastic wave velocity of Q345, *v*_1_ is the material point velocity of TA2 side, *v*_2_ is the material point velocity of Q345 side, $\sigma_{b}$ is the stress of the incident bar, $\varepsilon_{i}$ is the strain of the incident bar. Due to the existence of the welding interface, as long as the interface does not break, the interface no slip condition is met:

*v*_1_ = *v*_2_ = *v*_3_ (16)

where *v*_3_ is the material point velocity on the welding interface, substituting Equations (15) and (16) into Equation (14):

(17) $$v_{3}=\frac{E\varepsilon_{i}A_{0}}{\rho_{1}c_{1}A_{1}+\rho_{2}c_{2}A_{2}}$$

In the elastic stage, the internal stress $\sigma_{1}$ of TA2 side and $\sigma_{2}$ of Q345 side are:

(18) $$\sigma_{1}=\rho_{1}c_{1}v_{1}$$

(19) $$\sigma_{1}=\rho_{1}c_{1}v_{1}$$

Because of the wave impedance mismatch of TA2 and Q345, it must be impossible for the specimen to reach stress equilibrium in the elastic deformation stage, and there is shear stress at the interface:

(20) $$\sigma^{e}{}_{s}=\sigma_{1}-\sigma_{2}=\frac{\left(\rho_{1}c_{1}-\rho_{2}c_{2}\right)E\varepsilon_{i}{}^{e}A_{0}}{\rho_{1}c_{1}A_{1}+\rho_{2}c_{2}A_{2}}$$

where $\varepsilon_{i}{}^{e}$ is the strain corresponding to the elastic deformation stage in the strain-time curve of the incident bar. 

In the elastic deformation stage, the strain is uniform but the stress is not. As can be seen from Table 3, the time of elastic deformation stage of the specimen ranges from 0 to 6.2 μs, while the starting time of zone I is about 20 μs. This stage occurs before zone I in the *R*(*t*) − *t* diagram.

#### 3.4.2. Plastic Modulus Compatibility Deformation Stage

After the elastic deformation stage, the specimen entered the Plastic modulus compatibility deformation stage under the action of dynamic compression load. There may be three reasons for *R*(*t*) greater than 5% in zone I: First, it takes time for the stress wave to propagate in the specimen, and the stresses at both ends of the specimen are not equal in the initial response stage. The second reason is that the wave impedances of Q345 and TA2 are different. Under the condition of no slip at the interface, the stress distributions inside Q345 and TA2 are not uniform when the elastic deformation occurs. The axial stress distributions were found to be non-uniform in the elastic deformation range of the specimen. The third reason is that after the specimen enters the plastic deformation, it takes time for the welding interface to coordinate the internal stress of Q345 and TA2 from unequal to equal. Therefore, in the initial stage of plastic deformation, the stress at both ends of the specimen is unequal, but the plastic deformation is conducive to uniform stress distribution and transverse stress balance, while the plastic deformation is a tendency to produce a more homogeneous stress distribution within the components. The performance of *R*(*t*) in zone I can be deduced as the third one. Therefore, when the dynamic loading strain rate is low (such as in case 1), the specimen cannot quickly enter the plastic deformation stage, and *R*(*t*) cannot reach 5% at the end of zone I.

#### 3.4.3. Uniform Plastic Deformation Stage

According to regions II and III in Figure 7, the TA2/Q345 laminated specimen had a uniform stress deformation stage during the deformation process. Therefore, assuming that the internal stress on TA2 side is equal to that on Q345 side at this stage, it must be satisfied:

(21) $$\rho_{1}c_{p1}=\rho_{2}c_{p2}$$

where $c_{p1}$ and $c_{p2}$ are the plastic wave velocity of the titanium and the steel, respectively. According to the stress wave theory:

(22) $$c_{p}=\sqrt{\frac{E_{p}}{\rho }}$$

(23) $$E_{p}=\frac{d\sigma_{p}}{d\varepsilon_{p}}$$

where $c_{p}$ is the plastic wave velocity of the material, ρ is the density of the material, $\sigma_{p}$ is the plastic stress of the material, $\varepsilon_{p}$ is the plastic strain of the material and *E_p_* is the plastic modulus of the material. From Equations (21)–(23):

(24) $$\rho_{1}E_{p1}=\rho_{2}E_{p2}$$

where *E_p_*_1_ and *E_p_*_2_ are the plastic modulus of titanium and steel, respectively.

According to Equation (24), in this deformation stage, the welding interface plays a role in adjusting the plastic modulus of TA2 and Q345, so that the plastic stresses on both sides of the welding interface are equal, the specimens are uniformly deformed under the dynamic compression load, and the shear stress of the welding interface is zero at this stage. At this stage, the strain is uniform, and the stress is balanced during the deformation of the specimen. Therefore, this stage is called the uniform plastic deformation stage, corresponding to regions II and III in Figure 7.

After the elastic deformation stage and before the uniform plastic deformation stage, due to the compatibility of the plastic moduli of TA2 and Q345 at the welding interface, it takes time for the plastic stress on both sides of the welding interface to be unequal to equal. At this time, *R*(*t*) gradually approaches 5%, from greater than 5% to less than 5%. This compatibility process is called the plastic modulus compatibility deformation stage and corresponds to zone I in Figure 7.

#### 3.4.4. Non-Uniform Plastic Deformation Stage 

In case 5, when the strain rate increases to 2250 s^−1^, the axial plastic deformation of the specimen is 0.62 mm. At this time, the microscopic state of the welding interface is shown in Figure 9. It can be seen that the specimen cracks along the welding interface, which indicates that when the loaded strain rate reaches a certain value, the shear stress at the welding interface exceeds the compatibility capacity and fails. In the process of load response, the specimen will enter the non-uniform deformation and plasticity stage after undergoing uniform deformation and plasticity stage. At this time, the strain is uniform and the stress is not uniform in the deformation process of the specimen. Shear stress occurs again at the interface. When the interface shear stress is greater than the bonding strength of the welding interface, the welding interface will be damaged. After the welding interface is damaged, the condition of no slip is no longer satisfied, and Q345 and TA2 are deformed, respectively, under the loading of stress waves. At this time, the strain and stress of the specimen are not uniform during deformation.

Since the non-uniform deformation plastic stage occurs only when the loading strain rate is greater than a certain value and the time when this stage occurs is outside the range of experimental measurement (after 76.2 μs), the stress non-uniformity at this stage does not affect the stress–strain relationship obtained in the end.

### 3.5. Dynamic Compressive Mechanical Response of TA2/Q345

**Figure 10:**
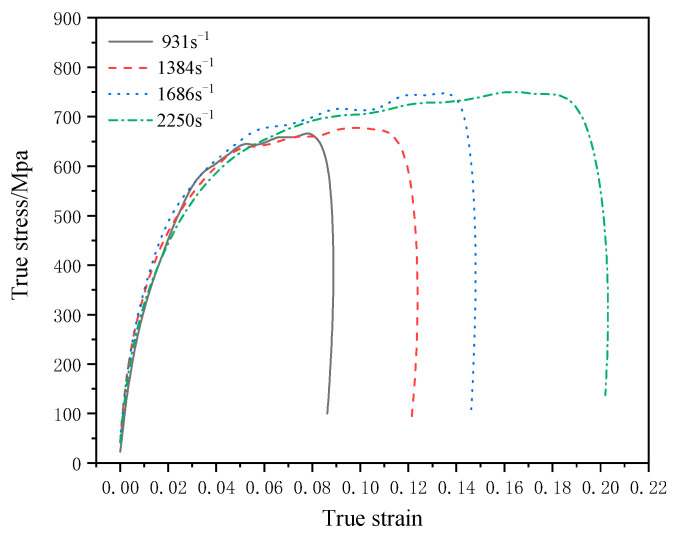
The true stress–strain curves of cases 2–5.

**Figure 11:**
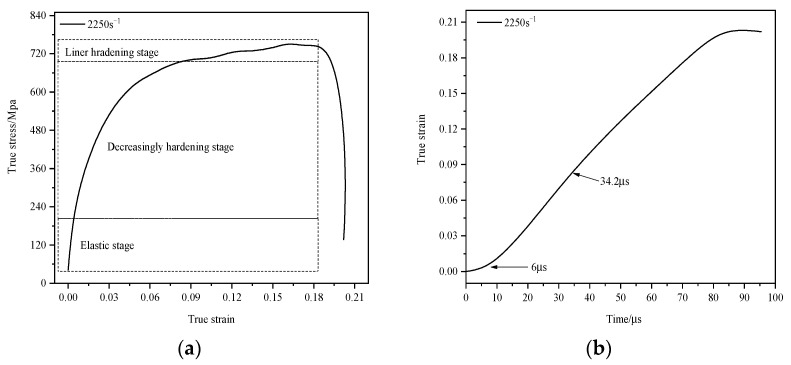
The true stress–strain curve (**a**) and the true strain–time curve (**b**) of case 5.

According to Equations (6) and (7), the nominal stress–strain curves of specimens under different strain rates can be obtained, and the nominal stress–strain curves can be converted into true stress–strain curves by simple conversion. The true stress–strain curves of specimens under different strain rates are shown in Figure 10. It can be seen from Figure 10 that the true stress–strain relationship under the loading conditions of four strain rates of cases 2–5 presents a consistent change tendency. In order to further analyze the dynamic compressive mechanical behavior of the specimen, the true stress–strain curve and the corresponding true strain–time curve of case 5 were plotted separately for analysis. The results are shown in Figure 11a and 11b, respectively.

As can be seen from Figure 11a, the true stress–strain curve can be divided into three stages. These are the elastic stage, the decreasing hardening stage, and the linear hardening stage, respectively. The initial deformation stage of the specimen is elastic. When the stress reaches 204.87 Mpa, the specimen begins to yield. Combined with the true strain–time curve (Figure 11b), the corresponding yield moment is 6. The specimen enters the plastic deformation stage after yielding. At the initial stage of plastic deformation, the plastic modulus of the specimen presents a decreasing hardening phenomenon, and at the late stage of plastic change, it presents a linear hardening phenomenon. The starting time of the linear hardening stage is 34.2 μs, corresponding to the starting time of zone II, indicating that the decreasing hardening stage is the plastic modulus compatibility stage, corresponding to zone I of the *R*(*t*) − *t* curve, and the linear hardening stage corresponds to zone II and zone III, indicating that the linear hardening stage is the uniform plastic deformation stage. The true stress–strain response of the specimen is consistent with the theoretical analysis of specimen deformation in Section 3.3.

In order to further analyze the dynamic compressive mechanical response behavior of TA2/Q345 laminated materials under parallel structure, the key node information in the true stress–strain curve under different strain rates was sorted into a table, and the results were shown in Table 3.

As can be seen from Table 3, with the increase of strain rate from 931 s^−1^ to 2250 s^−1^, the yield strength of TA2/Q345 laminates increases from 141.26 Mpa to 204.87 Mpa. It can be seen that TA2/Q345 laminated materials have a certain strain rate strengthening effect. TA2/Q345 laminated materials can be considered as strain rate-sensitive materials in the strain rate range of 931 s^−1^–2250 s^−1^. According to Figure 11, in the elastic stage, the stress increases sharply with the increase of strain, indicating that TA2/Q345 laminated materials have obvious strain hardening effect. With the increasing strain rate, the plastic strain of TA2/Q345 laminates increases significantly. At 931 s^−1^, the plastic strain is 0.08, and at 2250 s^−1^, the plastic strain increases to 0.18. The plastic fluidity of TA2/Q345 laminates is significantly enhanced, reflecting the obvious strain rate plasticizing effect. The reason for this strain rate plasticizing effect is that the specimen is in an adiabatic state during the deformation process of high strain rate, and the thermal energy generated by the impact load causes the temperature of the specimen to rise rapidly, softening the specimen and thus increasing the plastic fluidity of the specimen [29,30]. In the linear hardening stage, the true stress–strain curve presents periodic fluctuations, and strain hardening and strain softening alternately occur. This phenomenon may be caused by the competition between the strain hardening effect and the specimen softening effect caused by the adiabatic temperature rise [31].

## 4. Conclusions

The mechanism of deformation compatibility of parallel structure TA2/Q345 laminated materials is investigated using a 14.5 mm diameter SHPB system with strain rates in the range of 146 s^−1^~2250 s^−1^. The laminated material is fabricated using the explosive welding method. The welding interface was analyzed in detail before and after different dynamic compression loads. By analyzing the stress evolution law, the following conclusions are obtained:

1. The relative non-uniformity of the internal stress is lower than 5% when the strain rates are in range of 931–2250 s^−1^, indicating that the stress–strain relationships of the parallel structure specimens are reliable. The experimental results are real and effective. When the strain rate is at a relatively low level, i.e., strain rate is 146 s^−1^, the one-dimensional stress wave hypothesis and the stress uniformity hypothesis are not satisfied until the latter half of the effective loading time of the incident wave.
2. There exist four deformation stages of the parallel structure TA2/Q345 laminated material under the dynamic compression loading condition, namely, the elastic deformation stage, the plastic modulus compatible deformation stage, the uniform plastic deformation stage and the non-uniform plastic deformation stage. During the whole loading process, the proportion of the elastic deformation stage is only 7.5%. The plastic modulus compatible deformation stage accounts for 15.0%, mainly depending on the stress wave propagating in the specimen. The uniform plastic deformation stage accounts for the highest proportion of 53.7%, which is the primary stage in the process. The non-uniform plastic deformation stage happened with obvious characteristic of failure welding interface.
3. The plastic modulus-compatible deformation stage is characterized by the decreasing hardening phenomenon, while the uniform plastic deformation stage is characterized by the linear hardening of the plastic modulus. In the non-uniform plastic deformation stage, adiabatic temperature dominates the material behavior, leading to the specimen softening effect.
4. The parallel structure TA2/Q345 composites exhibit strain rate hardening effect, strain rate strengthening effect and strain rate plasticizing effect under 931 s^−1^–2250 s^−1^ strain rate dynamic compression load, which can be utilized in protective structure in electronic packaging, vehicle collision avoidance system et al.

### Future Scope

The primary goal of the current research is to figure out the mechanism of deformation compatibility of laminated metal with a parallel structure. However, the current study’s findings are restricted to one-dimensional conditions. Consequently, more research in three-dimensions will be needed in the future on the use of laminated metal in engineering.

## Figures and Tables

**Table 1:** Emission pressure and the velocity of striker bar.

Cases	1	2	3	4	5
Velocity of striker bar (m/s)	6.31	13.63	16.73	19.24	22.03
Emission pressure (MPa)	0.22	0.25	0.35	0.48	0.60

**Table 2:** Materials’ parameters of TA2, Q345 and SHPB bars.

	Density (g/cm^3^)	Hardness HV	Elasticity Modulus (GPa)	Elastic Wave Velocity (m/s)	Poisson Ratio
Q345	7.83	≥160	246.18	5607.2	0.3
TA2	4.51	≥140	132.82	5426.8	0.33
SHPB bars	7.69	≥500	200	5100.0	0.3

**Table 3:** The key node information in the true stress–strain relationship of cases 2–5.

Strain Rate (s^−1^)	Yield Strength (Mpa)	Yield Strain (10^–3^)	Yield Time (μs)	Initial Stress of Strain Hardening Effect (Mpa)	Initial Time of Linear Hardening (μs)
931	141.26	2.98	6.2	597.92	29.2
1384	169.47	2.56	5.8	638.56	33.2
1686	179.78	2.95	6	673.37	32
2250	204.87	4.22	6	695.33	34.2

## Data Availability

Data are contained within the article.
